# Eating Disorders and Disordered Eating Behaviors Among Undergraduate Students in Pakistan as Measured by Eating Disorder Examination Questionnaire (EDE-Q)

**DOI:** 10.7759/cureus.59158

**Published:** 2024-04-27

**Authors:** Muhammad Hassnain Nasrullah, Muhammad Jawad Haider, Maria Arif, Muhammad Nouman Zahid, Sana Iftikhar, Shahid Mahmood, Mehwish Akhtar, Muhammad Ammad bin Ilyas

**Affiliations:** 1 Community Medicine, Allama Iqbal Medical College, Lahore, PAK; 2 Community Health Sciences, Allama Iqbal Medical College, Lahore, PAK

**Keywords:** pakistan, medical students, undergraduate students, binge eating disorder, atypical anorexia, bulimia nervosa, anorexia nervosa, disordered eating behaviours, eating disorders, ede-q

## Abstract

Objectives

Eating disorders (ED) are an emerging public health issue globally, especially in young adults studying at the undergraduate level. This study aims to assess the frequency of eating disorders, their types, and disordered eating behaviors among such students. Moreover, it aims to identify factors like weight concern, shape concern, eating concern, and restraint, along with assessing the shifting trend of BMI impact on eating disorders using a standardized Eating Disorder Examination Questionnaire (EDE-Q).

Methods

In this cross-sectional study, 400 undergraduate students (aged 18-25) from four public universities participated from July 2022 to November 2023. Data was collected using the Eating Disorder Examination Questionnaire (EDE-Q). The frequency of eating disorders was computed using SPSS version 27.

Results

Among the participants, 21.75% (n=84) were identified as having a score surpassing the clinical cut-off. This group comprised 5.5% males (n=22) and 16% females (n=64). The highest prevalence among the four subscales was observed in the Shape Concern subscale (10.5%). Objective binge episodes (19.3%) emerged as the most notable disordered eating attitude. Atypical anorexia nervosa accounted for 13.8% of different eating disorders, while disordered eating was noted in 19.5% (n=78) of individuals.

Discussion

This study offers critical insights into eating disorders among Pakistan undergraduate students, utilizing the EDE-Q 6.0. Disordered eating behaviors, particularly shape concern and objective binge eating, exhibit significant correlations with these disorders. Weight dissatisfaction emerges as a prominent predictor, suggesting societal influence. The study also reveals a moderate correlation between BMI and eating disorders, challenging conventional assumptions. Furthermore, a changing trend in the prevalence of eating disorders is observed among the male population.

## Introduction

Eating disorders (ED) refer to a variety of psychological illnesses associated with significant disturbances in eating attitudes and behaviours. These may involve insufficient or excessive food intake related to an individual’s physical and emotional health. Young adults and those aged 18-25 years (college and university students) are susceptible to peer pressure, image-building pressure, and social media influences, and they are more likely to adopt unhealthy eating behaviour [1]. The most common forms of eating disorders are anorexia nervosa, bulimia nervosa, and binge eating. Anorexia nervosa is characterized by low body weight, intense fear of weight gain, and distorted perception of weight [2]. Bulimia nervosa, on the other hand, is a psychiatric condition characterized by recurrent consumption of large amounts of food in a short span followed by self-induced vomiting, fasting, overexercise, and/or misuse of laxatives/enemas or diuretics [3]. Binge eating disorder is similar to bulimia nervosa as far as consumption of a large amount of food, but consumption is in regular episodes, in rapid space of time, and secretly; the person may not be hungry, but there is an intense desire to overeat. However, these episodes of eating are followed by feelings of depression, shame, guilt, and low mood [4]. Despite the rising occurrence and severity of these disorders among young adults, only a few studies have examined the factors related to these disorders in developing countries like Pakistan.

Eating disorders are an emerging issue globally. Studies have reported a gradual increase in disordered eating behaviours in Western societies and some non-Western countries, with similar symptoms observed across these countries [3]. However, research on the clinical presentation of eating disorders (EDs), morbidity, and mortality remains largely focused on Western populations such as the United States, United Kingdom, Australia, and Western European countries [4]. What is of concern regarding the lack of research from non-Western settings is that many countries have gone through sociocultural changes. This acculturation process has been shown to impact psychological well-being and eating behaviours [5]. Eating disorders are complex illnesses that affect adolescents with increasing frequency [6]. Pakistan is considered to be the 5th country with the largest young population in the world [7]. Hence, it is of interest to explore the presentation of EDs in non-Western cultures, such as Pakistan, an under-researched culture that hosts one of the youngest populations in the world.

As of now, there is limited data on eating disorders in Pakistan, with only a handful of published reports available. These studies have used inventories such as the Eating Attitude Test 26 (EAT 26) [8] and the Body Shape Questionnaire (BSQ) [9]. In a study using the Eating Attitude Test 26, 495 undergraduate medical students responded. Of these, 22% were found to be at high risk of developing ED [8]. This indicates concerning levels of clinically significant ED symptoms among young people in Pakistan. Although these findings are relevant, no previous study in Pakistan has administered gold-standard symptom-reporting/diagnostic tools like the Eating Disorder Examination or the Eating Disorder Examination Questionnaire [10], which may limit potential comparisons with data from other countries where these measures are commonly used. The current study used the EDE- Q tool which is closest to the gold standard EDE Interview. There are four subscales: weight concern, shape concern, eating concern, and restraint. Data on these sub-scales are presented in this cross-sectional study for comparisons.

## Materials and methods

Following approval from the Ethical Review Board of Allama Iqbal Medical College/Jinnah Hospital, Lahore in its 130th meeting dated 03-11-2022, with Ethical Clearance Number I-2-2022, 400 students aged 18-25 from four different public universities were included in this cross-sectional study. A random sampling approach was made. Sample size calculation was based on taking 5% absolute precision at a 95% confidence interval and with the anticipated prevalence of 22.75% [8]. The minimum sample size needed was 270; nonetheless, time and availability enabled us to expand the sample to 400 to accommodate for missing data and no response, and facilitate calculations.

Eligibility was determined based on age, ranging from 18 to 25 years, and university enrollment, with exclusions made for clinically diagnosed medical and psychiatric conditions potentially associated with eating disorders. Those with a history of depressive disorder or generalized anxiety disorder were excluded. Participants completed the self-administered Eating Disorder Examination Questionnaire version 6.0 (EDE-Q 6.0) [11] from July 2022 through December 2023 after giving informed consent. EDE-Q 6.0 is a self-reported 28-question instrument with four domains that measure the range and severity of eating disorder behaviors and cognitions, including frequency of self-reported eating key and compensatory behaviors (objective binge-eating episodes, self induced vomiting, laxative misuse, and excessive exercise) over the previous 28 days. Scores are assigned ranging from 0 to 6, with 0 indicating no days of eating symptoms and 6 indicating symptoms experienced each day. EDE-Q 6.0 measured eating disorders in four scales: restraint, eating concerns, weight concerns, and shape concerns. Questions 1-5 measure restraint; questions 7, 9, 19, 20, and 21 deal with eating concerns; questions 8, 12, 22, 24, and 25 measure weight concerns; and questions 6, 8, 11, 23, 27, 28 measure shape concerns. The average of these four subscales (domains) was then used to calculate the final overall EDE-Q global score.

We have used the American Psychiatric Association Diagnostic and Statistical Manual of Mental Disorders, Fifth Edition, Text Revision (DSM-5TR) criteria [12], corresponding to particular questions within the EDE-Q, to determine various types of eating disorders presented in Table 1.

**Table 1:** Summary of eating disorders definitions based on DSM-5TR criteria and EDE-Questionnaire All the questions are extracted from EDE-Q 6.0 and formulated using DSM-5TR criteria. DSM-5TR: Diagnostic and Statistical Manual of Mental Disorders, Fifth Edition, Text Revision EDE-Questionnaire: Eating Disorder Examination Questionnaire

Eating Disorder	Criteria
Dietary restriction [13]	Going for long periods (≥8 hours) without eating anything to influence shape or weight for ≥13 days over the past 28 days (Question 2).
Excessive exercise [13]	Exercising in a driven or compulsive way as a means of controlling weight, shape, amount of fat, or burning off calories for ≥20 days over the past 28 days (Question 18).
Binge eating disorder [12]	Presence of objective binge-eating (Question 15 score ≥4) with no other compensatory behaviors.
Bulimia nervosa [12]	Objective binge eating (Question 15 score ≥4) with at least one compensatory behavior occurring at least once per week over the last 28 days (Questions 16, 17, or 18 score ≥4).
Anorexia nervosa [12]	Low body weight (BMI <17.5 kg/m2) with deliberate food intake limitation to influence weight or shape (Question 1 score ≥1) for ≥1 day over the past 28 days. Either weight (Question 22 score ≥4) or shape (Question 23 score ≥4) influences personal self-judgment.
Other specified feeding or eating disorders [12]	a) Atypical anorexia nervosa: Same guidelines as anorexia nervosa but BMI >17.5 kg/m2. b) Low frequency bulimia nervosa: Low frequency binge eating (Question 15 score 1-3) with no other compensatory behaviors. c) Low frequency binge eating disorder: Presence of low frequency binge eating score 1-3 (Question 15) only, with no other compensatory behaviors. d) Purging disorder: Self-induced vomiting (Question 16 score ≥4), laxative use (Question 17 score ≥4), and dietary restraint (Question 1 ≥3) with no other eating disorders.
Unspecified feeding or eating disorder [12]	Global score ≥4 with no other eating disorders diagnosed.

Studies show that the EDE-Q global score clinical cut-off of 1.68 for men has a sensitivity of 0.77 and specificity of 0.77, according to a study published in 2018 [14], and the global score clinical cut-off of 2.8 for women has a sensitivity of 0.80 and specificity of 0.80 [15].

All forms were checked for inconsistencies, and a unique identification number was allocated before data entry. The global score was calculated by averaging the score of all four subscales. We used Pearson’s correlation coefficient to assess the correlation among the global score, its various subscales, BMI, and disordered Eating behaviors.

SPSS version 27 was used for data management and analysis. Quantitative data, including the computation of mean with standard deviation and difference of mean, was analyzed. Categorical data was analyzed using percentages and differences of proportion. A p-value less than 0.05 was considered statistically significant.

## Results

A total of 400 undergraduate students with an average age of 20.83 years (SD=1.746), a mean BMI of 21.30 kg/m² (SD=3.89), and a response rate of 88.89% (n=355) participated in the study. Demographic characteristics of participants are presented in Table 2.

**Table 2:** Demographic characteristics of participants The data has been represented as %, n and Mean±SD, whereas total participants = 400. BMI: Body Mass Index

Physical Characteristics (Mean ± S.D)		
	Age	20.82±1.74 years
	Height	164±11.35 cm
	Weight	57.55±12.42 kg
BMI (Mean ± S.D)		
	Under weight <18.5 kg/m2	16.79±1.47 kg/m2
	Healthy weight BMI is 18.5 to <25 kg/m2	21.46±1.86 kg/m2
	Over weight BMI is 25.0 to <30 kg/m2	27.28±1.33 kg/m2
	Obese BMI is 30.0 kg/m2 or higher	31.88±2.11 kg/m2
Accommodation % (n)		
	Boarders	63 (252)
	Day Scholars	37 (148)
Examination System % (n)		
	Semester Exam System	56 (224)
	Annual Exam System	44 (176)
Student types % (n)		
	Arts Students	10 (40)
	Medical Students	30 (120)
	Engineering Students	20 (80)
	Business Students	25 (100)
	Computer Science Students	15 (60)

**Figure 1:**
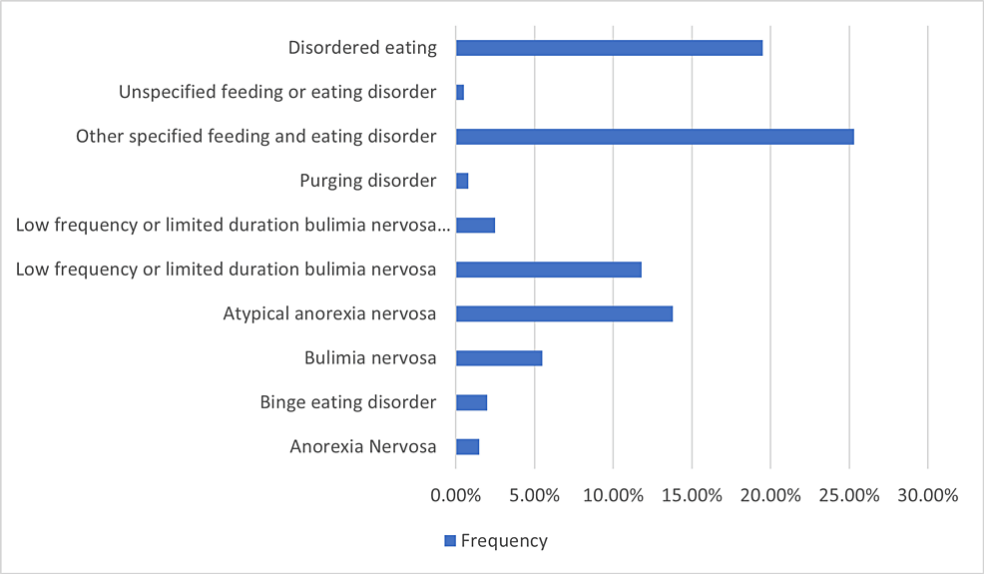
Eating disorders and disordered eating diagnosis using criteria of DSM-5TR guidelines. The data has been represented as %, whereas total participants = 400. DSM-5TR: Diagnostic and Statistical Manual of Mental Disorders, Fifth Edition, Text Revision

BMI distribution among the students categorized them as underweight (23.5%, n=94), healthy weight (63%, n=252), overweight (10.3%, n=41), and obese (3.3%, n=13). Among 400 participants, 12% (n=48) of medical students and 9.5% (n=38) of non-medical students (Arts, Computer Sciences, Engineering, and Business students) crossed the global score clinical cut-off. Eating disorders and disordered eating diagnosis using DSM-5TR [12] guidelines are explained in Figure 1.

The severity of overall and individual eating disorder psychopathologies measured using the EDE-Q global score and subscales among university attending students with BMI categorization are outlined in Table 3.

**Table 3:** Mean and Standard Deviation of the EDE-Q subscales for undergraduate students by body mass index (BMI) classification (N= 400) The data has been represented as Mean±SD. GS: Global score, EC: Eating Concern subscale, WC: Weight Concern subscale, SC: Shape Concern subscale, SD: Standard Deviation, EDE-Q: Eating Disorder Examination Questionnaire

	BMI Categories							
Subscales	Underweight<18.5 kg/m2		Healthy Weight BMI is 18.5 to <25 kg/m2		Overweight BMI is 25.0 to <30 kg/m2		Obese BMI is 30.0 kg/m2 or higher	
	Mean	SD	Mean	SD	Mean	SD	Mean	SD
Restrain	0.81	1.36	1.37	1.52	2.58	1.73	1.89	1.27
EC	0.84	0.98	1.11	1.10	1.93	1.56	1.40	1.11
SC	1.10	1.01	1.81	1.39	3.33	1.58	3.13	1.21
WC	1.21	1.11	1.65	1.42	3.03	1.59	2.71	1.34
GS	0.99	0.91	1.49	1.15	2.72	1.38	2.28	0.72

Notably, 8.8% (n=35) of participants scored in the clinically significant range (i.e., score ≥4) on the Restraint subscale, 3% (n=12)on the Eating Concern subscale, 10% (n=40) on Weight Concern subscale, 10.5% (n=42) on Shape Concern subscale, and 4% (n=16) on the Global Score subscale.

When comparing means of global scores and its subscales, participants with overweight BMI had significantly elevated mean global scores (2.72), dietary restraint (2.58), shape concern (3.33), weight concern (3.03), and eating concern (1.93) when compared to other categories of BMI in Table 3. Still, when pondering upon the disordered eating behaviours frequency, we find the healthy weight category to have a higher frequency than any other categories shown in Table 4.

**Table 4:** Proportion (%) of undergraduate students engaging in disordered eating behaviours, by body mass index (BMI) classification (N= 400) The data has been represented as %, n, whereas total participants = 400. BMI. Body Mass Index

		BMI Categories			
Disordered eating behaviour		Underweight<18.5 kg/m2	Healthy Weigh BMI 18.5 to <25 kg/m2	Overweight BMI 25.0 to <30 kg/m2	Obese BMI 30.0 kg/m2 or higher
Dietary restrictions	Any occurrence % (n)	2.30 (9)	14.50 (58)	3.80 (15)	1.00 (4)
	Regular occurrence % (n)	2.30 (9)	8.00 (32)	3.30 (13)	1.30 (5)
Objective binge episodes	Any occurrence % (n)	3.00 (12)	14.80 (59)	3.30 (13)	0.80 (3)
	Regular occurrence % (n)	3.00 (12)	14.00 (56)	2.00 (8)	0.30 (1)
Self-Induced vomiting	Any occurrence % (n)	2.30 (9)	4.30 (17)	0.50 (2)	0.30 (1)
	Regular occurrence % (n)	0.80 (3)	2.80 (11)	1.00 (4)	0.00 (0)
Laxative’s misuse	Any occurrence % (n)	1.80 (7)	4.30 (17)	0.30 (1)	0.30 (1)
	Regular occurrence % (n)	1.30 (5)	3.00 (12)	0.50 (2)	0.00 (0)
Excessive exercise	Any occurrence % (n)	4.50 (18)	17.30 (69)	3.00 (12)	0.30 (1)
	Regular occurrence %(n)	1.00 (4)	6.00 (24)	0.80 (3)	0.50 (2)

The prevalence of eating disorders among undergraduates came out to be 21.5% with a Global Score clinical cut-off of 2.8 for women and 1.68 for men (n=400) among which 5.5% (n=22) were males and 16% (n=64) were females. Among individuals crossing the clinical cut-off for the global score, 12% (n=48) had an annual examination system, while 9.5% (n=38) had a semester-based examination system. EDE-Q descriptive statistics by BMI category are shown in Tables 3 and 4.

**Figure 2:**
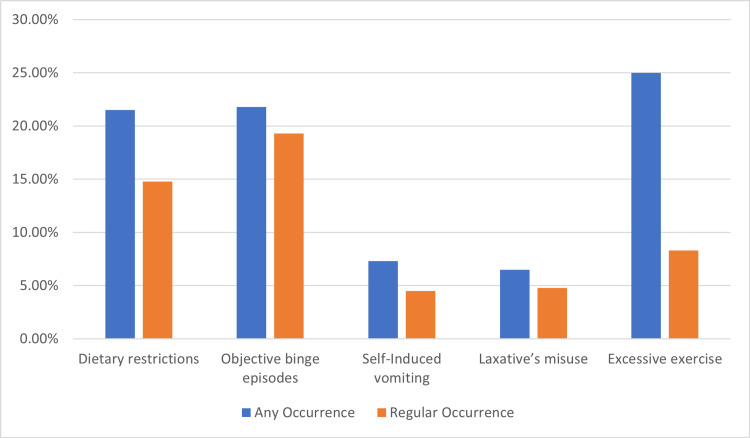
Proportion of undergraduate students engaging in disordered eating behaviours (N = 400) The data has been represented as %, where total participants = 400.

Any occurrence and regular occurrences of key ED behavioural features and Compensatory behaviours are presented in Figure 2.

Approximately 21.5% (n=86) of the individuals were reported to have any Occurrence of dietary restriction. 21.8% (n=87) were reported to have any occurrence of objective binge episodes. 7.2% (n=28) for self-induced vomiting and 6.5% (n=26) For laxative misuse. In comparison, a whopping 25% (n=100) were for excessive exercise within 28 days.

From Table 5, we can appreciate the strongest correlations between variables Shape Concern and Weight Concern (r = 0.886, p < 0.01), Global Score and Shape Concern (r = 0.920, p < 0.01).

**Table 5:** Associations between EDE-Q subscales, disordered eating behaviours, and BMI *. Correlation is significant at the 0.05 level (p> 0.05) BMI: Body Mass Index; GS: Global Score subscale; EC: Eating Concern subscale; WC: Weight Concern subscale; SC: Shape Concern subscale

	Restrain	EC	SC	WC	GS	BMI	Dietary restrictions	Objective binge episodes	Self-Induced vomiting	Laxative’s misuse	Excessive exercise
Restrain											
EC	0.43*										
SC	0.58*	0.66*									
WC	0.57*	0.67*	0.89*								
GS	0.78*	0.78*	0.92*	0.92*							
BMI	0.32*	0.25*	0.49*	0.42*	0.44*						
Dietary restrictions	0.66*	0.28*	0.32*	0.35*	0.48*	0.24*					
Objective binge episodes	0.08	0.30*	0.20*	0.21*	0.22*	0.05	0.02				
Self-Induced vomiting	0.14*	0.18*	0.14*	0.18*	0.19*	-0.01	-0.03	0.12*			
Laxative’s misuse	0.13*	0.22*	0.13*	0.17*	0.19*	0.005	0.10*	0.32*	0.32*		
Excessive exercise	0.30*	0.26*	0.26*	0.24*	0.31*	0.12*	0.09	0.29*	0.16*	0.22*	

When considering the Global Score as a reference, among the subscales of the Global Score, the highest correlation, which is statistically significant, is of the Shape Concern variable. Similarly, among the demographic information, the highest correlation with the Global Score is BMI. While moving towards disordered eating behaviors, the highest correlation was dietary restriction followed by excessive exercise. These strong correlations suggest that higher concerns about body image, weight, and eating behaviours are associated with elevated overall eating disorder scores. Similarly, increased restraint in eating behaviours is linked to higher overall eating disorder scores.

## Discussion

This study presents a statistical analysis utilizing the EDE-Q 6.0, providing vital descriptive statistics on eating disorders among Pakistan undergraduate students.

We report that the prevalence of eating disorders as measured by the EDE-Q global score is 21.5% of a 400 undergraduate student sample. Of this, 21.5%, 16% were females, and 5.5% were males. This is comparable to a cross-sectional study carried out in Karachi, Pakistan [8] that reported 22.75% of its sample population of undergraduate medical students to likely have been suffering from eating disorders, although that study used EAT 26; our study utilizes the EDE-Q in this region, offering valuable insights into eating disorders and disordered eating behaviors. This substantiates the fact that eating disorders mount a significant burden on the health of undergraduates.

The subscale which represents the strongest correlation of the Global Score is the Shape Concern subscale (r = 0.920, p < 0.01). Similarly, Virginia et al. [16] reported similar findings in their 2012 study on US college students with the Shape Concern subscale having the highest mean value (r = 2.39, p < 0.01). These findings are consonant with the fact that undergraduate students are especially conscious about their physical appearance. They often feel compelled to compete with peers at their universities about keeping their bodies in an appealing state. It is also pertinent to pin this on the increased use of social media in recent times, as images on these platforms promote an ideal body figure [17].

Objective binge eating was the most commonly reported disordered eating behavior, with a regular occurrence frequency of 19.3%. This is consistent with several studies conducted in the past on undergraduates [16,18,19]. One plausible explanation for this is the ‘hurried lifestyle’ many students develop at university while trying to juggle a lot.

The Shape Concern variable had the highest correlation with the Global Score (r=0.920), emphasizing its strong association with eating disorders. Additionally, dietary restriction demonstrated a strong positive correlation (r=0.488) with the Global Score, highlighting the significant impact of societal influences, particularly from social media [17], on the development of eating disorders in young individuals.

In the regression analysis exploring the influence of various questions in the EDE-Q on the Global Score, dissatisfaction with weight (Question 25) emerged significantly, with an odds ratio of 1.99. This underscores the societal influence of the idealized body image [17], doubling the likelihood of weight dissatisfaction and the development of eating disorders.

Our study reflects a moderate correlation (r=0.440) between BMI with Global Score. This means that, unlike the general perception that maintaining the body weight within the supposed ‘healthy range’ is certainly protective of developing an eating disorder, it is not right. This is further corroborated by the fact that the most commonly occurring specified eating disorder in our study, found using the DSM-5TR criteria [12] about questions in the EDE-questionnaire, is atypical anorexia nervosa, i.e., anorexia nervosa in people with a BMI equal to or greater than 17.5 kg/m2. We can also appreciate this relation when we look at Table 3, which shows the highest frequency of disordered eating behaviors among the healthy weight category.

Eating disorders tend to occur more frequently among female undergraduate students, their prevalence being 28.32% in our study compared to 25% for men. This, compared to previous studies, shows an increasing prevalence of eating disorders among the male population of society [8]. Similarly, all of the disordered eating behaviours are more common among women than men. This could be explained by women trying to conform to the idealistic thin female body perceived to be sexually desirable and of good health [4], posing as risk factors for disordered eating.

There is a slightly higher prevalence of eating disorders among undergraduate medical students (12%) compared to undergraduates enrolled in other non-medical sciences (Arts, CS, Engineering, Business) programs (9.5%). A more hectic day-to-day routine, a more general expectation to perform well, and an annual system of examination with a cumbersome load of syllabi to be assessed for medical students explains this higher prevalence. Moreover, certain medications such as laxatives are more accessible to such students.

Comparing different factors, such as the type of study (medical vs. non-medical) and examination systems (annual vs semester), we observed a 3.5% difference in the percentage of the population with the clinical cut-off for the global score between medical students with annual examination system and non-medical students with semester-based examination system, suggesting a potential influence of the type of study and examination system on the development of eating disorders. Despite our study's strengths, including the utilization of EDE-Q 6.0 in the Pakistan's demographic context and a well-executed random sampling method, there are limitations to our study. We only included four teaching institutes in Lahore, limiting the generalizability of our findings to the entire population of Pakistan.

Our study focused on undergraduates attending universities in an urban region of the country. This remains a great limitation in applying the findings more broadly to the general public. Also, consideration for the type of boarding, rural population, economic status, and hometown is required. Lastly, we relied on clinical cut-offs from other countries due to the lack of specific clinical cut-offs for Pakistan. Future research needs to establish precise clinical cut-offs for the diagnosis of eating disorders using the EDE-Q 6.0 in the Pakistan population. Overall, our study provides valuable insights into the prevalence and factors associated with eating disorders among undergraduate students in Pakistan, but further research is needed to address the limitations and enhance the understanding of this complex issue in our demographic context.

Public significance

This cross-sectional study employs the EDE-Q 6.0 to investigate eating disorders among Pakistan undergraduates, highlighting factors influencing diagnosis and associated disordered eating behaviors. The findings offer valuable insights for potential revisions to DSM-5TR guidelines in this demographic. Additionally, the study underscores the impact of BMI and academic program type on eating disorders among undergraduate students, contributing to a deeper understanding of this complex issue in the Pakistani context.

## Conclusions

Our study concluded with significant numbers that nearly a quarter of undergraduate students are likely to have been suffering from eating disorders while most of the remaining students have disordered eating, potentially leading to the development of eating disorders. Students with an annual examination system in medical studies are more prone to developing eating disorders than students with semester-based examinations in non-medical studies (Arts, CS, Engineering, Business). Also, most of the individuals having a higher frequency of disordered eating behaviors fall into the healthy BMI category, pointing towards the shifting trend of eating disorders etiology. Strikingly, in contrast to previous studies, there seems to be an increasing prevalence of eating disorders among the male student population, now reaching nearly to that of their female counterpart.

## References

[1] de Matos AP, Rodrigues PR, Fonseca LB, Ferreira MG, Muraro AP (2021). Prevalence of disordered eating behaviors and associated factors in Brazilian university students. Nutr Health.

[2] Castillo M, Weiselberg E (2017). Bulimia nervosa/purging disorder. Curr Probl Pediatr Adolesc Health Care.

[3] Gunewardene A, Huon GF, Zheng R (2001). Exposure to westernization and dieting: a cross-cultural study. Int J Eat Disord.

[4] Scharff A, Ortiz SN, Forrest LN, Smith AR (2021). Comparing the clinical presentation of eating disorder patients with and without trauma history and/or comorbid PTSD. Eat Disord.

[5] Thomas J (2013 ). The new Arabia felix. Psychological Well-Being in the Gulf States.

[6] (1998). Eating disorders in adolescents: Principles of diagnosis and treatment. Paediatr Child Health.

[7] Hafeez E, Fasih T (2018). Growing population of Pakistani youth: A ticking time bomb or a demographic dividend. J Educ Educ Dev.

[8] Memon AA, Adil SE, Siddiqui EU, Naeem SS, Ali SA, Mehmood K (2012). Eating disorders in medical students of Karachi, Pakistan-a cross-sectional study. BMC Res Notes.

[9] Suhail K, Zaib-u-Nisa Zaib-u-Nisa (2002). Prevalence of eating disorders in Pakistan: relationship with depression and body shape. Eat Weight Disord.

[10] Fairburn CG, Beglin SJ (1994). Assessment of eating disorders: interview or self-report questionnaire?. Int J Eat Disord.

[11] Fairburn GC, Bohn K (2008). Eating disorder examination questionnaire. Cognitive behavior therapy and eating disorders.

[12] American Psychiatric A (2022). Diagnostic and Statistical Manual of Mental Disorders.

[13] Pirotta S, Barillaro M, Brennan L (2019). Disordered eating behaviours and eating disorders in women in Australia with and without polycystic ovary syndrome: A cross-sectional study. J Clin Med.

[14] Schaefer LM, Smith KE, Leonard R (2018). Identifying a male clinical cutoff on the Eating Disorder Examination-Questionnaire (EDE-Q). Int J Eat Disord.

[15] Mond JM, Myers TC, Crosby RD (2008). Screening for eating disorders in primary care: EDE-Q versus SCOFF. Behav Res Ther.

[16] Quick VM, Byrd-Bredbenner C (2013). Eating Disorders Examination Questionnaire (EDE-Q): norms for US college students. Eat Weight Disord.

[17] Vandenbosch L, Fardouly J, Tiggemann M (2022). Social media and body image: recent trends and future directions. Curr Opin Psychol.

[18] Sahlan RN, Taravatrooy F, Quick V, Mond JM (2020). Eating-disordered behavior among male and female college students in Iran. Eat Behav.

[19] Nakai Y, Nin K, Fukushima M (2014). Eating disorder examination questionnaire (EDE-Q): norms for undergraduate Japanese women. Eur Eat Disord Rev.

